# Myocardium at risk in ST-elevation myocardial infarction: comparison of T2-weighted edema imaging with the endocardial surface area assessed by magnetic resonance and validation against angiographic scoring

**DOI:** 10.1186/1532-429X-13-S1-O31

**Published:** 2011-02-02

**Authors:** Georg F Fuernau, Ingo Eitel, Vinzenz Franke, Lysann Hildebrandt, Josefine Meissner, Matthias Gutberlet, Steffen Desch, Gerhard C Schuler, Holger Thiele

**Affiliations:** 1 University of Leipzig - Heartcenter, Leipzig, Germany

## Objectives

T2-weighted magnetic resonance imaging (MRI) and the endocardial surface area (ESA) assessed by late gadolinium enhanced (LGE) have been introduced as relatively new methods for myocardium at risk assessment in ST-elevation myocardial infarction (STEMI).

## Background

However, data on the utility and validation of these techniques are limited. Purpose was therefore to assess the area at risk (AAR) with these 2 MRI methods and to compare them to the validated angiographic APPROACH-score in a large consecutive patient cohort.

## Methods

One-hundred-ninety-seven patients undergoing primary percutaneous coronary intervention (PCI) in acute STEMI were included. AAR (assessed with T2-weighted edema imaging and the ESA method), infarct size and myocardial salvage were determined by MRI 2-4 days after primary PCI. Angiographic AAR scoring was performed by use of the APPROACH-score.

## Results

The AAR assessed by T2-weighted imaging showed a good correlation with the angiographic AAR (r=0.87; p<0.001), whereas the ESA showed only a moderate correlation either to T2-weighted imaging (r=0.56; p<0.001) and the APPROACH-Score (r=0.44; p<0.001). Mean AAR by ESA (20.0±11.7%LV) was significantly smaller than AAR by T2-weighted imaging and APPROACH-Score (p<0.001 respectively) and showed a significant negative dependence on myocardial salvage index.

## Conclusion

Myocardium at risk and subsequently the salvaged AAR can be reliably assessed by T2-weighted edema imaging, whereas assessment of the AAR by ESA seems to be dependent on myocardial salvage and underestimates the AAR especially in patients with high myocardial salvage and aborted infarction. Thus, assessment of the AAR with the ESA method cannot be recommended.

